# Ferroptosis, a Potential Therapeutic Target in Alzheimer’s Disease

**DOI:** 10.3389/fcell.2021.704298

**Published:** 2021-08-05

**Authors:** Kai Chen, Xiaobing Jiang, Moxin Wu, Xianming Cao, Wendai Bao, Ling-Qiang Zhu

**Affiliations:** ^1^Key Lab of Neurological Disorder of Education Ministry, Department of Pathophysiology, School of Basic Medicine, Tongji Medical College, Huazhong University of Science and Technology, Wuhan, China; ^2^Department of Neurosurgery, Wuhan Union Hospital, Tongji Medical College, Huazhong University of Science and Technology, Wuhan, China; ^3^Department of Jiujiang Clinical Research Center for Precision Medicine, Affiliated Hospital of Jiujiang University, Jiujiang, China; ^4^College of Biomedicine and Health, Huazhong Agricultural University, Wuhan, China

**Keywords:** ferroptosis, cell death, iron, lipid peroxidation, AD

## Abstract

Cell death is a common phenomenon in the progression of Alzheimer’s disease (AD). However, the mechanism of triggering the death of neuronal cells remains unclear. Ferroptosis is an iron-dependent lipid peroxidation-driven cell death and emerging evidences have demonstrated the involvement of ferroptosis in the pathological process of AD. Moreover, several hallmarks of AD pathogenesis were consistent with the characteristics of ferroptosis, such as excess iron accumulation, elevated lipid peroxides, and reactive oxygen species (ROS), reduced glutathione (GSH), and glutathione peroxidase 4 (GPX4) levels. Besides, some ferroptosis inhibitors can relieve AD-related pathological symptoms in AD mice and exhibit potential clinical benefits in AD patients. Therefore, ferroptosis is gradually being considered as a distinct cell death mechanism in the pathogenesis of AD. However, direct evidence is still lacking. In this review, we summarize the features of ferroptosis in AD, its underlying mechanisms in AD pathology, and review the application of ferroptosis inhibitors in both AD clinical trials and mice/cell models, to provide valuable information for future treatment and prevention of this devastating disease.

## Introduction

**FIGURE 1:**
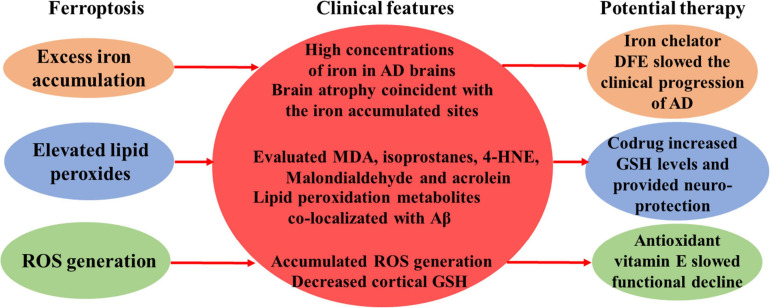
Clinical features of ferroptosis in AD.

Ferroptosis is a recently defined iron-dependent form of cell death induced by the small molecule erastin (Dixon et al., 2012). This newly described process possesses different morphological, biochemical and genetical features, which is distinct from apoptosis, necrosis, autophagy, and other forms of cell death (Weiland et al., 2019). The classic features of ferroptosis include the specifical changes of cellular morphology, the iron-dependent accumulation of lipid peroxides, and ROS, the depletion of GSH, and inactivation of GPX4, and a distinct set of regulated genes (Dixon et al., 2012; Dixon, 2017; Chen et al., 2021). Additionally, ferroptosis could be specifically inhibited by a series of inhibitors, such as iron chelators deferoxamine (DFO) and lipid peroxidation inhibitors ferrostatin-1 (Fer-1), but not sensitive to apoptotic or necroptotic inhibitors (Dixon et al., 2012). Notably, signatures of ferroptosis, such as excess iron accumulation, elevated lipid peroxides, and ROS generation have long been discovered in brains of AD patients and model mice (Zhang et al., 2012; Hambright et al., 2017; Ayton et al., 2019). Moreover, some ferroptosis inhibitors, such as desferrioxamine (DFE) and vitamin E have exhibited clinical benefits in AD

(Mclachlan et al., 1991; Dysken, 2014; Figure 1). Therefore, these findings strongly indicated the involvement of ferroptosis in the pathogenesis of AD. However, the direct evidence of ferroptosis in AD pathology is still lacking. In this article, we will systematically review the current knowledge of ferroptosis in AD, discuss the underlying mechanism, and describe the possible therapeutic strategies to treat this disease.

The clinical features of AD which were consistent with the characteristics of ferroptosis were noted here, including high concentrations of iron in AD brains, brain atrophy coincident with the iron accumulated sites, elevated lipid peroxidation products, such as MDA, isoprostanes, 4-HNE, malondialdehyde and acrolein, and clinical benefits of some ferroptosis inhibitors.

## Characteristics of Ferroptosis in AD

### Excess Iron Accumulation

Ferroptosis is an iron-dependent cell death form and emerging evidences have reported that iron overload could directly induce ferroptosis *in vivo* and *in vitro* under pathological process (Wang et al., 2017; Fang et al., 2019). In the central nervous system, iron is an essential element that is involved in many important biological processes in brain, such as oxygen transportation, myelin production, and the synthesis of neurotransmitters (Ward et al., 2014). Several studies have observed the selective accumulation of iron in the Aβ aggregation and neurofibrillary tangles of AD brains (Good et al., 1992; Tao et al., 2014), and the excess brain iron accumulation was associated with accelerated cognitive decline in AD patients (Ayton et al., 2019). Moreover, high concentrations of iron which was detected by immune-histochemical staining (Xian-hui et al., 2015; Sands et al., 2016) and MRI analysis (Schenck et al., 2006; Langkammer et al., 2014), are accumulated in various brain regions [including temporal cortex (Ayton et al., 2019), hippocampus (Schenck and Zimmerman, 2004; Antharam et al., 2012), caudate nucleus and the basal ganglia (Langkammer et al., 2014)] and associated with the motor and cognitive impairments of AD pathology. Furthermore, age-related iron accumulation contributed to the tissue damage and pathologic manifestations of AD (Raven et al., 2013). Collectively, these findings indicate that excess iron accumulation is a common pathological feature in the course of AD.

Brain atrophy is a prominent feature of AD and reduced volumes of medial-temporal part of brain, especially the hippocampus was frequently reported in the brains of AD patients, which was closely correlated with the impaired global cognitive performance (de Jong et al., 2008; Uysal and Ozturk, 2020). Moreover, the diffuse cortical atrophy could also be observed in patients with abnormal cognitive scores (Ten Kate et al., 2018). Interestingly, the brain regions which suffered neuronal degeneration and brain atrophy were highly coincident with the sites of iron accumulation (Horvath et al., 2012), these findings strongly suggest that the excess iron accumulation is linked to interferonopathies of AD. On the other hand, iron is an important component of the catalytic subunit of lipoxygenase (LOX) that catalyzes the oxygenation of polyunsaturated fatty acids (PUFAs) (Kuhn et al., 2015). It could generate the ROS through the donation of electrons to oxygens, and promote the ferroptosis (Stockwell et al., 2017). Thus, the perturbed iron distribution in AD probably induced or enhanced the ferroptosis, which could contribute to neuronal death and degeneration in this disease.

### Elevated Lipid Peroxides and ROS

Accumulation of massive lipid peroxides to lethal levels was another characteristic of ferroptosis, and the biomarkers of lipid peroxides were also elevated in the AD pathology. Clinically, lipid peroxidation metabolites showed co-localization with the amyloid plaques, and were highly related to the AD progression (Benseny-Cases et al., 2014). For early and non-invasive diagnosis, various studies were done to evaluate the lipid peroxidation products, such as malondialdehyde (MDA) (Krishnan and Rani, 2014), isoprostanes (Peskind et al., 2014), 4-Hydroxynonenal (4-HNE) (Di Domenico et al., 2017), malondialdehyde and acrolein (Williams et al., 2006; Di Domenico et al., 2017), as the diagnostic markers in the onset of AD. Among these candidates, MDA, isoprostanes, and 4-HNE were most promising and consistent between different studies (Pena-Bautista et al., 2019). These findings indicated that the accumulated lipid peroxides also contribute to the neuropathology of AD, and some lipid peroxidation products can act as the biomarkers for AD diagnosis and prognosis.

Besides the increased lipid peroxides, accumulated lipid ROS and decreased cortical GSH content also have been found in the AD pathology. Previous studies reported that excess ROS is generated under pathological AD conditions and reducing ROS accumulation could restore cognition in AD model rats (Smith et al., 2010; Wang et al., 2016). Moreover, the levels of membrane phospholipids [phosphatidylethanolamine (PE) and phosphatidylinositol (PI)]-derived total fatty acids were significantly decreased in the hippocampus of AD patients (Prasad et al., 1998), and AD-associated reductions of GSH levels also have been observed in both animal models (Resende et al., 2008; Zhang et al., 2012) and the human brains (Chiang et al., 2017). Notably, the levels of GSH showed a close relationship with the brain amyloidosis and AD pathology. Overall, all of these evidences implicated that lipid oxidative stress, the key process of ferroptosis, is also intimately involved in the pathological development of AD.

## Ferroptosis-Related Signaling Pathways in AD

**FIGURE 2:**
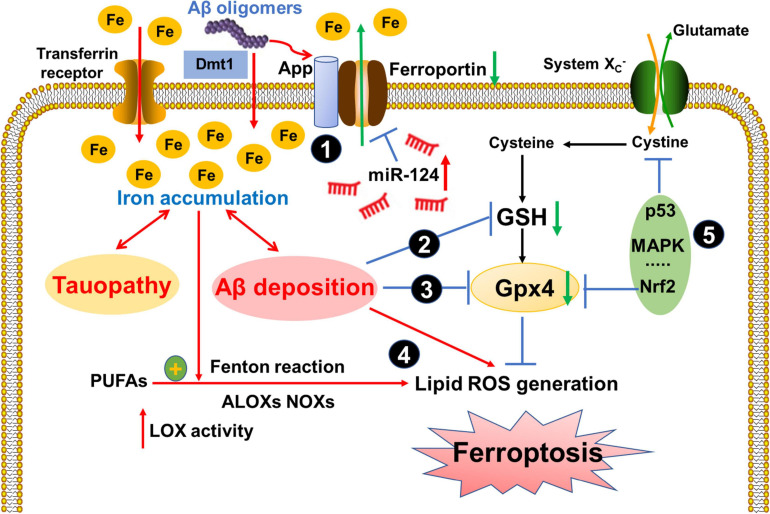
Ferroptosis-related signaling pathways in AD.

Ferroptosis in AD has been extensively studied in the past decades and there are multiple signaling pathways were reported in the regulation of ferroptotic cell death in AD, such as the perturbed iron export and transport in iron metabolism pathway, the reduced GSH and Gpx4 levels in redox homeostasis pathway, the accumulated lipid peroxidation and ROS generation in lipid metabolism pathway and some other potential signaling pathways, as summarized in Figure 2. In the following text, we will discuss the mechanism of each ferroptosis-related signaling pathway in AD.

Signaling pathways that may be pathologically involved in the ferroptosis in AD. (1) APP ferroxidase activity is inhibited and Ferroportin (Fpn) is reduced in the AD brain, which inhibited the iron export and resulted in the excess iron accumulation. Moreover, the levels of miR-124, which directly induced a posttranscriptional deregulation of Fpn, also increased in AD patients; (2) Iron accumulation aggravated toxic Aβ deposition and tau hyperphosphorylation, which disrupted GSH synthesis and reduced Gpx4 levels (3); (4) Elevated LOX catalyzed PUFAs and generated ROS via Fenton reaction. Additionally, Aβ deposition also led to lipid peroxidation and ROS generation. (5) Other pathways, such as p53, Nrf2, MAPK etc.

### Iron Metabolism Pathway

Iron dysregulation, one of the hallmarks of ferroptosis, have been implicated in AD pathogenesis for a long time. Iron dyshomeostasis has been reported to aggravate toxic amyloid-β (Aβ) deposition and hyperphosphorylated tau aggregation, the two main histopathological features of AD. Indeed, tau hyperphosphorylation and aggregation also contribute to toxic neuronal iron accumulation, which leads to a vicious cycle (Yan and Zhang, 2019). Moreover, rats fed with high iron-diet demonstrated an increased Aβ deposition and tau hyperphosphorylation, and iron chelator effectively attenuated Aβ load and tau aggregation (Sripetchwandee et al., 2014). Similarly, Tau deficiency impairs amyloid-beta precursor protein (APP)-mediated iron export and this change can be prevented by a moderate iron chelator, clioquinol (Lei et al., 2012). Taken together, these studies suggest that iron dysregulation plays a deleterious role in AD and targeting iron metabolism pathway may be an effective way for manipulating ferroptosis in AD.

Iron export and iron transport are the two aspects of maintaining iron homeostasis. However, the expression levels of two major iron-exporters, ferroportin (Fpn) and iron-homeostatic peptide hepcidin, were both reported to be reduced in the AD brain (Raha et al., 2013). Moreover, the APP ferroxidase activity, which could interact with iron-exporter Fpn and facilitate the removal of cytoplasmic iron, also was inhibited in AD postmortem neocortex (Duce et al., 2010). Interestingly, the levels of miR-124, which directly induced a posttranscriptional deregulation of Fpn is also increased in AD patients, future confirming the deregulation of Fpn in AD (Wang et al., 2018; Bao et al., 2020). Accordingly, a recent study indicated that deficiency of Fpn in principal neurons in neocortex increased iron levels and induced AD-like hippocampal atrophy and memory deficits, while restoring Fpn expression could effectively ameliorate the memory loss and ferroptosis in AD model mice (Bao et al., 2021). Besides the perturbed iron export, the expression levels of divalent metal transporter 1 (Dmt1), the main transporter of divalent metal ions which is colocalized with the Aβ in AD pathology, were significantly increased in the AD transgenic model and aged murine model (Xian-hui et al., 2015; Tian et al., 2018). Interestingly, silencing of endogenous Dmt1 could reduce iron accumulation and lead to reduction of Aβ secretion (Zheng et al., 2009). Additionally, transferrin receptor protein 1 (Tfr1), the major vehicle of iron delivery into cells, also was significantly upregulated in AD mice. Treatment with the active compounds of herbs ameliorate impaired cognition and this beneficial effect is associated with reduced impairment of iron metabolism (Yu et al., 2019; Ma et al., 2021). Thus, enhancing iron export or inhibiting iron transport could be a promising strategy to reduce the ferroptotic cell death in this disease.

### Redox Homeostasis Pathway

Redox homeostasis is a vital component of a healthy physiological steady state. GSH is the major cellular antioxidant which maintaining redox homeostasis from the following two aspects: bind to ferrous iron in the labile iron pool (LIP) to prevent the iron-dependent oxidization (Hider and Kong, 2011) and as the substrate of GPX4-mediate lipid detoxification (Friedmann Angeli et al., 2014; Yang et al., 2014). Numerous studies have shown that alterations of GSH content impaired redox homeostasis and are associated with the occurrence of ferroptosis in AD (Pocernich and Butterfield, 2012; Hambright et al., 2017). Casley et al. (2002) reported that addition of Aβ could induce depletion of GSH in cultured neurons. Inversely, administration of GSH precursor gamma-glutamylcysteine ethyl ester (GCEE) increased cellular GSH levels and protected against Aβ-mediated neurotoxicity in neuronal cells (Boyd-Kimball et al., 2005). Moreover, AD-associated reductions of brain GSH levels have been observed during AD onset and progression *in vitro* (Ghosh et al., 2012) and *in vivo* (Resende et al., 2008). These findings strongly indicated that disruption in GSH homeostasis is associated with AD pathology. In addition to alterations in GSH levels, GSH-associated antioxidant enzyme glutathione S-transferases (GSTs), which catalyze the reaction between GSH and 4-HNE, was also changed in AD pathology (Aquilano et al., 2014). Both the enzyme activity and protein levels of GSTs were significantly decreased in most brain regions and cerebrospinal fluid (CSF) in AD patients (Lovell et al., 1998). Moreover, glutathione S-transferase omega-1 and 2 genes (GSTO1, GSTO2) polymorphisms have been implicated as risk factors for earlier onset of AD (Allen et al., 2012). Thus, restoring GSH levels would be an effective way of attenuating AD progression. Indeed, some researches have focused on finding potential approaches for restoring GSH levels in AD patients (Bermejo et al., 2008; Cacciatore et al., 2012).

In addition to the disruption of GSH synthesis, inhibition of Gpx4 was another vulnerability factor for ferroptosis-related AD pathogenesis (Friedmann Angeli et al., 2014). Gpx4 is a member of an antioxidant enzyme family which catalyze the reduction of hydroperoxides by GSH or other biological reductants (Cardoso et al., 2017). This family was composed of eight isoenzymes (Gpx1-Gpx8) and Gpx4 is the most widely expressed one in brain (Wang et al., 2013). Previous study has reported that Gpx4 was identified as the key regulatory enzyme in ferroptosis of AD (Yang et al., 2014). Ablation of Gpx4 in forebrain, the frequently affected region in AD pathology, leads to cognitive impairment and hippocampal neurodegeneration in Gpx4 brain inducible knockout (Gpx4BIKO) mice. Moreover, the classical features of ferroptosis, such as elevated lipid peroxidation, extracellular signal-regulated kinase (ERK) activation and amplified neuroinflammation, were also observed and treatment with a ferroptosis inhibitor could ameliorate neurodegeneration in those mice (Hambright et al., 2017). Besides the inhibition of Gpx4 enzyme activity, the expression of guanine-rich sequence-binding factor (Grsf1), which controls the translation of Gpx4, were also downregulated in brain of AD model mice (Yoo et al., 2010). Moreover, polymorphisms in Gpx1 and Gpx4 were significantly associated with memory impairment and AD in a South Brazilian population (da Rocha et al., 2018). Collectively, these findings demonstrate that decreased GSH content and reduced Gpx4 levels lead to redox imbalance, targeting this redox homeostasis pathway could help alleviate ferroptosis-related damage in AD.

### Lipid Metabolism Pathway

Science ferroptosis is a lipid peroxidation-driven cell death form, the abundance and localization of PUFAs, which determine the degree of lipid peroxidation, also was crucial for the execution of ferroptosis in AD (Stockwell et al., 2017). Previous study revealed a co-localization of lipid peroxidation products and Aβ plaques in the brain of AD patients (Butterfield and Boyd-Kimball, 2018). Butterfield reported that Aβ peptides led to lipid peroxidation. In turn, lipid peroxidation products increase APP processing (Butterfield, 2020). Noticeably, lipoxygenase (LOX), cyclooxygenases (COXs), and cytochrome p450 (CYPs), three well-defined classes of lipid oxidation enzymes which catalyze the deoxygenation of PUFAs, have been reported were changed in AD pathology (Shintoku et al., 2017; Wu et al., 2018). Di Meco et al. (2017) reported that the enzymatic activity of 12/15-lipoxygenase (12/15-LOX) was significantly increased in the brains of AD patients, and its protein levels influence the memory and learning abilities in mouse model. Importantly, pharmacological inhibition of 12/15-LOX can reverse this AD-like phenotype (Di Meco et al., 2017). Therefore, the significant dysregulation of lipid peroxidation and perturbed lipid metabolism suggested an early involvement of this pathway in AD pathophysiology.

Sphingosine kinase1 (Sphk1), which could acetylate cyclooxygenase 2 (COX2), also were downregulated in the brains of AD patients and AD mice. Restoration of the expression of Sphk1 could promote the acetylation of COX2 and improve the AD-like pathology in APP/PS1 mice (Lee et al., 2018). Sphk2, another neuronal sphingosine kinase, also have been reported to protect against hippocampal volume loss and demyelination in male J20 mice (Lei et al., 2019). Additionally, a fatty acid synthase inhibitor, CMS121, protects against ferroptosis-induced lipid peroxidation and alleviates cognitive impairment in APP/PS1-transgenic mice (Ates et al., 2020). Taken together, these findings demonstrated that restoration of lipid peroxidation effectively rescued the AD-like symptoms and lipid metabolism pathway will be a potential therapeutic target for the prevention of this disease in the future.

### Other Pathways

Besides these directly involved signal pathways, a series of molecules that mediating the cell protection and neuronal death may also regulate the occurrence of ferroptosis in AD pathogenesis. Emerging evidences implicated that p53, a critical regulator of cell cycle and cell death, directly participate in the induction of ferroptosis (Jiang et al., 2015; Gnanapradeepan et al., 2018; Zhang W. et al., 2018). Jiang et al. (2015) demonstrated that p53 repressed expression of SLC7A11, the key component of the cystine/glutamate antiporter, thus inhibiting cystine uptake and sensitizing tumor cells to ferroptosis. Interestingly, p53 were also reported to play a pivotal function in oxidative stress-triggered neuronal death in the progression of AD (Cenini et al., 2008). However, whether p53 could directly regulate the ferroptosis in AD still need to be further explored. Similar to these reports, the activation of mitogen-activated protein kinase (MAPK) signaling pathway also were observed in both ferroptosis induction (Dixon et al., 2012) and AD pathological process (Kheiri et al., 2018), probably indicating the involvement of this signaling pathway in the ferroptosis in AD.

A key transcriptional regulator, nuclear factor erythroid2-related factor 2 (Nrf2), which containing the antioxidant response elements (ARE) and targeting the antioxidant effectors (etc., HO-1 and GSH), also was involved in the ferroptosis in AD (Kerr et al., 2017). Kerr et al. (2017) reported that Aβ inhibit Nrf2 activity in *Drosophila*, whereas specific inhibition of Keap1, the negative regulator of Nrf2, can rescue Nrf2 activity and ameliorate Aβ-induced neuronal toxicity. Many other studies also demonstrated that genetic ablation of Nrf2 in APP/PS1 mice promotes AD-like pathology (Joshi et al., 2015; Ren et al., 2020). Importantly, Nrf2 was thought to participate in the process of ferroptosis on account of its function in glutathione, iron, and lipids metabolism (Dodson et al., 2019). Although the underlying mechanism was still elusive, abundant evidence suggested the protective roles of Nrf2 in ferroptosis and neurodegeneration (Abdalkader et al., 2018; Bahn and Jo, 2019). Considering the critical role of Nrf2 in ferroptosis-related damage in AD, some Nrf2 inducers could also be efficaciously therapeutic agents for this disease.

## Application of Ferroptosis Inhibitors in AD

### Ferroptosis Inhibitors in AD Clinical Trials

Emerging evidences suggested that ferroptosis is a potential therapeutic target for AD. Some ferroptosis inhibitors have already showed possible clinical benefits in clinical trials. A two year, single-blind study which administration of iron chelator DFE or oral placebo (lecithin) in 48 patients with probable AD. The researchers found that DFE treatment led to significant reduction in the rate of deterioration in AD, and the mean rate of decline was twice as rapid for the no-treatment group. These results revealed the potential role of ferroptosis inhibitor DFE in slowing the clinical progression of the dementia associated with AD (Mclachlan et al., 1991). Moreover, in a large randomized clinical trial, another low potency anti-ferroptotic agent, vitamin E, also exhibited clinical benefits in patients with mild-moderate AD. The benefits of vitamin E in mild to moderate AD by slowing functional decline and decreasing caregiver burden (Dysken, 2014; Dysken et al., 2014). Collectively, these findings suggested the potential clinical benefits of ferroptosis inhibitors in mild to moderate AD and application of ferroptosis inhibitors may slow the progression of this disease.

### Ferroptosis Inhibitors in AD Cell and Animal Models

Ferroptosis inhibitors also showed the protective roles in AD cell and animal models. A recent study found that Fer-1 and liproxstatins-1(Lip-1), two ferroptosis inhibitors, could effectively ameliorate the neuronal death and memory loss induced by Aβ aggregation *in vitro* and *in vivo* (Bao et al., 2021). A naturally occurring iron chelator, α-Lipoic acid (LA), effectively rescued tauopathy and cognitive impairment in P301S Tau transgenic mice. Interestingly, Tau-induced iron overload and lipid peroxidation, which are involved in ferroptosis, were significantly blocked by LA administration. They also found that LA improve these abnormalities by reducing ROS content and increasing the expression level of GPX4 (Zhang Y. et al., 2018). Futuremore, selenium (Se), the key regulator of GPX4 activity, has been considered to be related to AD pathologies (Chmatalova et al., 2017; Vinceti et al., 2017). Supplementation with organic form of Se, Se-enriched yeast (Se-yeast) or selenomethionine (Se-Met), could improve cognitive impairment and AD-related pathological symptoms in animal model of AD (Zhang Z. et al., 2018). Similarly, More et al. (2018) reported that N-acetylcysteine (NAC), an antioxidant and a glutathione precursor, attenuate the spatial memory deficits and synaptic plasticity loss by decreasing lipid oxidation and increasing GSH content in animal model of AD (da Costa et al., 2017; More et al., 2018). Another antioxidant, a nanomicellar water-soluble formulation of coenzyme Q10 (Ubisol-Q10), drastically inhibited Aβ plaque formation and improved long term memory (Muthukumaran et al., 2018). Taken together, these results indicate that ferroptosis may be an important neurodegenerative mechanism in AD and ferroptosis inhibitor could provide promising approach for treatment and prevention of this disease.

## Conclusion and Prospects

Alzheimer’s disease is a progressive brain degenerative disease which is the most common form of dementia among older people. In this review, we provided comprehensive summarization about the potential involvement of ferroptosis in AD and discussed the underlying signaling pathways that were probably involved in the ferroptotic death of neuronal cells during the pathogenesis of this disease.

Furthermore, we also summarized the ferroptosis inhibitors that have been applicated in the treatment and prevention of this disease (Table 1). The discovery of ferroptosis and the potential regulatory signaling pathways in this article, provide not only novel insights into the neuronal death in AD, but also trackable targets for this disease. Finally, ferroptosis also has been shown to implicate in the pathological cell death of several other neurologic diseases, such as Parkinson’s disease (PD), Huntington’s diseases (HD), amyotrophic lateral sclerosis (ALS), ischemic and hemorrhagic stroke, traumatic brain injury (TBI), and ischemia-reperfusion injury (Guiney et al., 2017; Stockwell et al., 2017; Weiland et al., 2019; Shen et al., 2020; Yan et al., 2021). Further research needs to be done to test the clinical benefits of ferroptosis inhibitors in these neurologic diseases.

**TABLE 1:** Summary of ferroptosis inhibitors that have been applicated in AD.

**Reagent**	**Target**	**Study models**	**Conclusions**	**References**
Deferoxamine (DFO)	Iron	Human cancer cells	DFO inhibited erastin-induced ROS accumulation and cell death	Dixon et al., 2012
Desferrioxamine (DFE)	Iron	Clinical patients	DFE slowed the clinical progression of the dementia associated with AD	Mclachlan et al., 1991
Clioquinol	Iron	Mouse model	Clioquinol prevented Tau deficiency impaired iron export	Lei et al., 2012
α-Lipoic acid (LA)	Iron	Mouse model	Effectively rescued tauopathy and cognitive impairment in P301S mice	Zhang Y. et al., 2018
Ferrostatin-1(Fer-1) Liproxstatin-1(Lip-1)	Lipid peroxidation	Primary neurons Mouse model	Fer-1/Lip-1 effectively ameliorate Aβ induced neuronal death and memory loss	Bao et al., 2021
Vitamin E	Lipid peroxidation	Clinical patients	Vitamin E slowed cognitive decline in patients with mild to moderate AD	Dysken et al., 2014
N-acetylcysteine (NAC)	Lipid peroxidation	Mouse model	Attenuated spatial memory deficits and synaptic plasticity loss in AD mice	More et al., 2018
Coenzyme Q10 (CoQ10)	Lipid peroxidation	Mouse model	Improved AD-Type behavioral and pathological symptoms in APP/PS1 mice	Muthukumaran et al., 2018
Selenium (Se)	Gpx4	Mouse model	Improved cognitive impairment and AD -related pathological symptoms in mice	Zhang Z. et al., 2018

## Author Contributions

L-QZ and WB conceived and designed the work. KC and XJ wrote the manuscript and revised it. KC, XC, and MW designed the table and figures. All authors contributed to the article and approved the submitted version.

## Conflict of Interest

The authors declare that the research was conducted in the absence of any commercial or financial relationships that could be construed as a potential conflict of interest.

## Publisher’s Note

All claims expressed in this article are solely those of the authors and do not necessarily represent those of their affiliated organizations, or those of the publisher, the editors and the reviewers. Any product that may be evaluated in this article, or claim that may be made by its manufacturer, is not guaranteed or endorsed by the publisher.
